# Genetic diversity of *Echinococcus granulosus *sensu lato from animals and humans in Bosnia and Herzegovina

**DOI:** 10.1186/s13071-022-05598-9

**Published:** 2022-12-08

**Authors:** Adnan Hodžić, Amer Alić, Amir Spahić, Josef Harl, Relja Beck

**Affiliations:** 1 Division of Microbial Ecology (DoME), Department of Microbiology and Ecosystem Science, Centre for Microbiology and Environmental Systems Science (CMESS), University of Vienna, 1030 Vienna, Austria; 2 Department of Parasitology and Invasive Diseases, Faculty of Veterinary Medicine, University of Sarajevo, 71000 Sarajevo, Bosnia and Herzegovina; 3 Department of Clinical Sciences of Veterinary Medicine, Faculty of Veterinary Medicine, University of Sarajevo, 71000 Sarajevo, Bosnia and Herzegovina; 4 Department of Pathology, Cantonal Hospital, 72270 Travnik, Bosnia and Herzegovina; 5 Department for Pathobiology, Institute of Pathology, University of Veterinary Medicine Vienna, 1210 Vienna, Austria; 6 Laboratory for Parasitology, Department for Bacteriology and Parasitology, Croatian Veterinary Institute, 10000 Zagreb, Croatia

**Keywords:** *atp*6, Bosnia and Herzegovina, *cox*1, *Echinococcus granulosus*, Genotypes, Intermediate hosts

## Abstract

**Background:**

Cystic echinococcosis (CE) is recognized as one of the most prevalent zoonotic diseases in Bosnia and Herzegovina. However, no systemic investigation of the genetic diversity of *Echinococcus granulosus* sensu lato circulating among animals and humans in the country has been performed to date.

**Methods:**

In this preliminary study, we analysed one cyst each from 36 sheep, 27 cattle, 27 pigs, 11 wild boars and 16 human patients for amplification and partial sequencing of the adenosine triphosphate 6 (*atp*6) and cytochrome *c* oxidase 1 (*cox*1) genes. The host species, fertility rate and organ cyst location were recorded for each subject involved in the study.

**Results:**

Overall, the *atp*6 gene was successfully amplified and sequenced from 110 samples, while 96 of the PCRs for *cox*1 were positive. Three zoonotic genotypes of *E. granulosus* sensu stricto (G1 and G3) and *Echinococcus canadensis* (G7) were identified in our isolates based on analyses of the *atp*6 gene. These genotypes were represented by 11 different genetic variants (haplotypes), six of which were identified for the first time in the present study.

**Conclusions:**

This study demonstrates, for the first time, that CE in Bosnia and Herzegovina is predominantly caused by *E. granulosus *sensu stricto and *E. canadensis* clusters, which exhibited a lower genetic diversity compared to isolates from other European countries. Further molecular studies employing other mitochondrial and nuclear genes are required to better understand the transmission cycles of *E. granulosus* sensu stricto among intermediate and definitive hosts in the country.

**Graphical Abstract:**

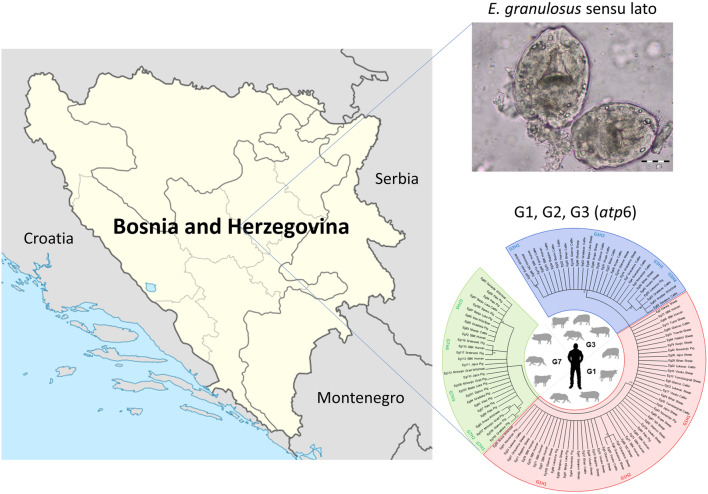

## Background

Cystic echinococcosis (CE) is a neglected zoonotic disease caused by the larval form (metacestode or CE cyst) of the dog tapeworm species cluster *Echinococcus granulosus *sensu lato (*E. granulosus* s.l.). Adults of this parasite inhabit the small intestine of dogs and other canids, while herbivores or omnivores serve as intermediate hosts in which the larval form develops following the ingestion of the parasite’s eggs released in faeces. Humans are accidental intermediate hosts. CE has a worldwide distribution and is endemic in many regions where livestock breeding is practiced, including the Mediterranean and Balkan countries (reviewed in [1, 2]). According to the Food and Agriculture Organization of the United Nations and WHO criteria, *E. granulosus* s.l. is one of the most important food-borne parasites for which WHO advocates control measures [3].

In the last decades, molecular studies principally based on mitochondrial DNA sequences have shown that *E. granulosus* is a complex of five cryptic species and several genotypes that differ in development rate, host specificity, pathology and sensitivity to chemotherapeutic drugs. These species include *Echinococcus granulosus *sensu stricto (*E. granulosus* s.s.) (genotypes [G] 1 and 3), *Echinococcus equinus* (G4), *Echinococcus ortleppi* (G5), *Echinococcus canadensis* (G6/7 and G8/10) and *Echinococcus felidis* (‘lion strain’) [4–8]. G2 has recently been recognized as a microvariant of G3 and is therefore no longer considered to be a separate genotype [9]. The taxonomic status of the *E. canadensis* cluster (G6/7, G8 and G10) is still under debate [10], and recently proposed names, i.e. *Echinococcus intermedius* for G6/7, *E. borealis* for G8 and *E. canadensis* for G10 [11], have not yet been widely accepted by the international scientific community [12].

To date, there has been very little information on CE in Bosnia and Herzegovina, with a few limited studies reporting sero-/prevalence of infection in humans and domestic animals or clinical case reports [13–16]. According to these studies, CE is recognized as the most important zoonotic disease in the country, with a prevalence ranging from 20% to 81% in cattle and sheep [16]. An epidemiological study conducted in 2002 revealed a seroprevalence of 8.3% among residents in Herzegovina (the southernmost part of the country) [16], while a considerably higher seroprevalence of 17.9% was reported in a later study [16], showing an increasing trend in the burden of CE in the country.

No information is as yet available on the genetic make-up of *E. granulosus* s.l. species and the genotypes circulating in Bosnia and Herzegovina. Therefore, this study aimed to molecularly characterize CE cysts collected from animals and humans and to examine the genetic variation and haplotype compositions of *Echinococcus* isolates in the country.

## Methods

### Study area

**Fig. 1:**
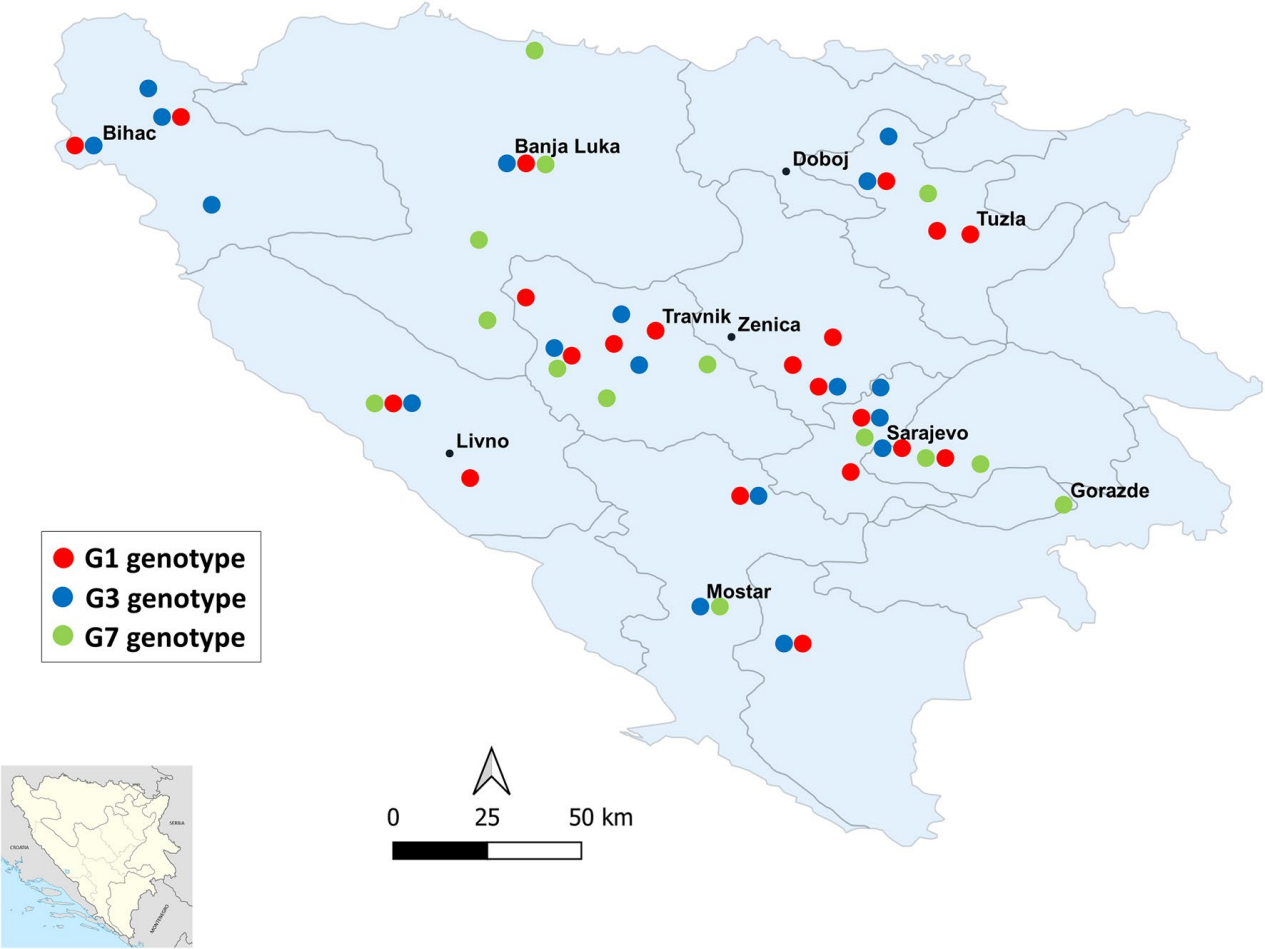
Map of Bosnia and Herzegovina showing the distribution of *Echinococcus granulosus* sensu lato genotypes identified in animals and humans. Map was created in QGIS 3.4 via QGIS.org (https://qgis.org/en/site/)

This study was carried out in Bosnia and Herzegovina, which covers 51,209.2 km^2^ and is situated in the western part of the Balkan Peninsula (43°52ʹ N, 18°25ʹ E; Fig. 1). The central and eastern part of the country is mountainous with a continental mountain climate, whereas the northeast is predominantly flat with a moderate continental climate. Herzegovina is characterized by dominant karst and plain topography with a typical Mediterranean climate. The fauna of Bosnia and Herzegovina is considered to be among the most diverse in Europe, mostly because of its ecological heterogeneity and geomorphologic, hydrological and eco-climate diversity [17].

### Collection of CE cysts

Samples from domestic animals were collected between 2012 and 2014 by veterinarians during routine meat inspections at abattoirs located across Bosnia and Herzegovina (Fig. 1). Infected organs from wild boars were retrieved and delivered by hunters (2012–2014, 2020). In addition, formalin-fixed paraffin-embedded (FFPE) tissue cysts collected from human patients who underwent surgery in the Cantonal hospital, Travnik (Central Bosnia Canton) were also included in this study. Overall, 117 CE cysts (one cyst per subject) were obtained from animals and humans (sheep, *n* = 36; cattle, *n* = 27; pig, *n* = 27, wild boar, *n* = 11; human, *n* = 16) and analysed by molecular tools. Data on organ location of the cysts and fertility were documented for each subject individually.

### Microscopic examination of the cyst fertility

The fertility of the collected cysts was assessed following centrifugation of the cysts’ content at 1500 *g* for 10 min followed by microscopic examination of the resulting sediment for the presence of protoscoleces. Protoscoleces (fertile cysts) or small pieces of the germinal layer (sterile cysts) of each cyst were stored in a plastic tube containing 70% alcohol until molecular analyses. The presence of protoscoleces in the FFPE samples was assessed by light microscopy on stained histological slides.

### DNA extraction and PCR

Before DNA extraction, collected protoscoleces and germinal layers were washed several times in sterile saline solution and dried at room temperature. Genomic DNA was extracted from each protoscolece and/or germinal layer individually using a DNeasy^®^ Blood and Tissue kit (Qiagen, Hilden, Germany) and an automatic extraction system (QIAcube^®^; Qiagen). One sample of DNase/RNase-free distilled water (Promega, Madison, WI, USA) was included in each extraction round as a blind control. The FFPE samples were deparaffinized with xylene and ethanol and subjected to DNA extraction using the same method. The DNA extracts obtained from the FFPE samples were additionally purified with a MinElute™ PCR Purification Kit (Qiagen).

A 674-bp fragment of the mitochondrial adenosine triphosphate 6 (*atp*6) gene was amplified using the primers *atp*6-F (5′-GCATCAATTTGAAGAGTTGGGGATAAC-3′) and *atp*6-F (5′-CCAAATAATCTATCAACTACACAACAC-3′) [18, 19]. In addition, the primers JB3 (5′-TTTTTTGGGCATCCTGAGGTTTAT-3′) and JB4.5 (5′-TAAAGAAAGAACATAATGAAAATG-3′) were used to amplify a 414-bp-long fragment of the cytochrome *c* oxidase 1 (*cox*1) gene [20]. PCR analyses were carried out in a final volume of 20 µl containing 10 µl G2 GoTaq^®^ G2 Mastermix (Promega), 7.2 µl of DNase/RNase-free distilled water (Promega), 0.4 µl of 10 pmol/µl of each primer and 2 µl of DNA template.

The amplification products were visualized by capillary electrophoresis (QIAexcel^®^ System; Qiagen) using a QIAxcel^®^ DNA Fast Analysis Kit and alignment markers (DNA QX Alignment Marker 15 bp/3 kb and QX DNA Size Marker 50–3000 bp; Qiagen).

### Sequencing and phylogenetic analyses

The amplified PCR products were purified using ExoSAP-IT™ PCR Product Cleanup Reagent (USB^®^ Products, Cleveland, OH, USA) according to the manufacturer’s instructions and then sequenced in both directions with the same primers used for the PCRs (Macrogen, Amsterdam, the Netherlands). The nucleotide sequences obtained were edited with BioEdit software v.7.2.5 [21] and compared for similarity with those available in GenBank^®^ using the Basic Local Alignment Search Tool (BLASTn) (http://www.ncbi.nlm.nih.gov/BLAST).

Phylogenetic analysis was performed only on a dataset completed with the longer *atp*6 nucleotide sequences of *E. granulosus* s.l. Multiple sequence alignment was performed with the ClustalW algorithm implemented in BioEdit v.7.2.5 [21], and the sequences were trimmed manually, so the overall alignment was 674 bp in length. A neighbour-joining (NJ) tree was constructed applying the TN93 model (according to second-order Akaike information criterion [AICc] values) implemented in the MEGA v.7.0 bioinformatics software [22]. The heuristic tree search and tree topologies algorithm reliability test were estimated with 1000 replicates.

The evolutionary distances between and within species/genotypes (*p*-distance) were calculated by MEGA v.7.0 [22]. The Flatworm mitochondrial code [23] was employed to infer amino acids from the nucleotide sequences.

### Statistical analysis

Proportions of CE cyst fertility and positivity rates between affected organs were compared with Fisher’s exact tests. Differences were considered statistically significant at *P* < 0.05. All statistical analyses were performed with GraphPad 5 Prism software (GraphPad Software Inc., San Diego, CA, USA).

## Results

### *Echinococcus granulosus* s.l. in Bosnia and Herzegovina

**Fig. 2:**
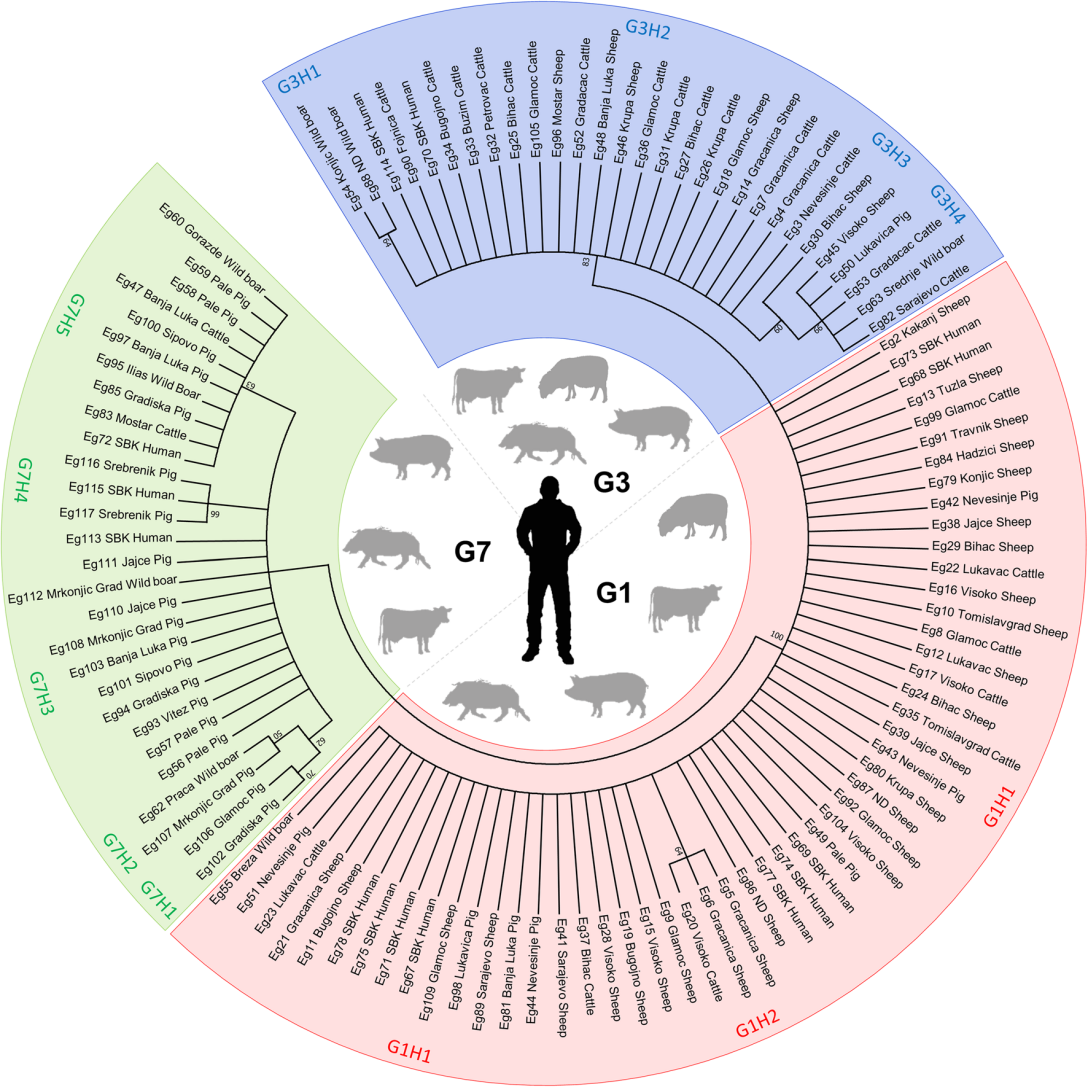
Neighbour-Joining bootstrap tree constructed with the adenosine triphosphate 6 gene (*atp*6) nucleotide sequences of *Echinococcus granulosus* sensu lato (674 bp). Bootstrap values based on 1000 replicates are indicated at the nodes (only values > 50% are included). The name, origin, and host species for each nucleotide sequence are shown. ND, no data; SBK, Central Bosnia Canton

The presence of CE cysts was most frequently detected in the liver (*n* = 81) and lungs (*n* = 32) of infected animals and humans, whereas the spleen (*n* = 3) and the kidneys (*n* = 1) were much less affected (Table 1). Of the 117 cysts examined, PCR amplification of *atp*6 and *cox*1 revealed positive results in 110 (94%) and 92 (78.6%) cases, respectively. *atp*6 was successfully amplified and sequenced from the majority of the FFPE samples collected (14/16; 87.5%), while *cox*1 was detected in only six of these same FFPE samples (37.5%). Notably, the PCR positivity rate was considerably higher when the FFPE DNA extracts were purified and then used as templates (3 vs 14 in the *atp*6 PCRs; 0 vs 6 in the *cox*1 PCRs). For further phylogenetic analyses, only *atp*6 nucleotide sequences were employed because of the higher PCR positivity rate and also because a recent study demonstrated that the *cox*1 gene marker is not sufficiently consistent for the differentiation between G1 and G3 genotypes [9], which is also in line with our findings. The phylogenetic analysis showed that almost half of the isolates belong to the highly pathogenic G1 genotype (*n* = 53; 48.2%) followed by the G3 (*n* = 29; 26.4%) and G7 (*n* = 28; 25.4%) genotypes (Table 1; Fig. 2). All animal species investigated in the present study were infected with all three genotypes, with the exception of sheep in which G7 was absent (Table 1; Fig. 2).

**Table 1:** Genetic diversity, fertility rate, and organ affinity of *Echinococcus granulosus* sensu lato isolates from Bosnia and Herzegovina

Species	Genotype (*n*)	Host (*n*)	Fertile:infertile (%)	Liver:lungs (%)
*Echinococcus granulosus *sensu stricto	G1 (53)	Sheep (28)	22:6* (78.6:21.4)	23:5** (82.1:17.9)
		Cattle (8)	3:5 (37.5:62.5)	6:2 (75.0:25.0)
		Pig (7)	7:0* (100.0:0.0)	4:3 (57.1:42.9)
		Wild boar (1)	1:0 (100.0:0.0)	0:1 (0.0:100.0)
		Human (9)	7:2 (77.8:22.2)	8:0* (100.0:0.0) + 1 spleen
	G3 (29)	Sheep (7)	5:2 (71.4:28.6)	3:4 (42.9:57.1)
		Cattle (16)	0:16** (0.0:100.0)	6:10 (37.5:62.5)
		Pig (1)	1:0 (100.0:0.0)	1:0 (100.0:0.0)
		Wild boar (3)	2:1 (66.7:33.3)	2:1 (66.7:33.3)
		Human (2)	1:1 (50.0:50.0)	1:0 (100.0:0.0) + 1 spleen
*Echinococcus canadensis*	G7 (28)	Cattle (2)	0:2 (0.0:100.0)	0:2 (0.0:100.0)
		Pig (19)	12:7 (63.1:36.9)	18:0*** (100.0:0.0) + 1 kidney
		Wild boar (4)	4:0 (100.0:0.0)	3:1 (75.0:25.0)
		Human (3)	1:2 (33.3:66.7)	2:0 (100.0:0.0) + 1 spleen

Significant difference at: *P < 0.05, **P < 0.001, ***P < 0.0001

### Haplotype composition of *E. granulosus* s.s. and *E. canadensis*

The *E. granulosus* s.s. G1 genotype was represented by only two haplotypes (designated as G1H1 and G1H2), with a single stepwise mutation difference (Fig. 2; Table 2). Overall, G1H1 was the most dominant genotype and showed a 100% identity with other, globally distributed G1 sequences [24]. The second G1 haplotype (G1H2), detected only in two sheep and one cow, had a 99.9% similarity to other *atp*6 G1 sequences mostly identified in humans from Europe and North Africa [24]. The NJ tree displayed the existence of four and five genetic variants among G3 and G7 sequences, respectively (Fig. 2; Table 2). G3H2 was the most commonly recorded haplotype within the G3 genotype and showed a 100% identity to the sheep, cattle and human isolates from Europe and camels from Iran [24]. The haplotype G3H1 was present in wild boars only. The G3H3 haplotype was represented by only one sequence (100% identity), which was previously identified in sheep and humans from Europe and Algeria [24]. Two haplotypes of G3 (G3H1 and G3H4) were characterized for the first time in the present study, with both showing a 99.9% similarity to the G3 sequences reported from animals and humans in Europe, North Africa and Iran [24].

**Table 2:** Differences in the mitochondrial adenosine triphosphate 6 gene *atp*6 gene sequences of representative *E. granulosus* sensu lato genotypes/haplotypes

Species	Genotype (*n*)	Haplotype (*n*)	Point mutations										
			17	78	155	320	343	382	424	433	461	540	646
*Echinococcus granulosus *sensu stricto	G1 (53)	G1H1 (50)	G	·	·	·	T	·	T	T	G	T	T
		G1H2 (3)	A	·	·	·	T	·	T	T	G	T	T
	G3 (29)	G3H1 (2)	G	·	·	·	T	·	C	C	A	T	C
		G3H2 (21)	G	·	·	·	T	·	C	T	A	T	C
		G3H3 (1)	G	·	·	·	T	·	C	T	A	C	C
		G3H4 (5)	G	·	·	·	C	·	C	T	A	C	C
*Echinococcus canadensis*	G7 (28)	G7H1 (2)	·	T	T	T	·	T	·	·	·	·	·
		G7H2 (2)	·	C	T	T	·	T	·	·	·	·	·
		G7H3 (11)	·	C	T	G	·	T	·	·	·	·	·
		G7H4 (3)	·	C	T	G	·	C	·	·	·	·	·
		G7H5 (10)	·	C	C	G	·	T	·	·	·	·	·

New haplotypes (H) are underlined. Dots indicate sites where sequences are not depicted to the representative *E. granulosus* sequence due to different genotypes

Genotype G7 of *E. canadensis* showed a greater haplotype diversity amongst our isolates (Fig. 2; Table 2). Of the five G7 haplotypes characterized in the present study, three were unique to Bosnia and Herzegovina (G7H1, G7H4 and G7H5), with a 99.9% similarity to the G7 isolates from pigs, wild boars and sheep in Europe [24]. The sequences of the G7H3 haplotype had a 100% identity and were almost exclusively found in pigs and wild boars in our study and the study of Kinkar et al. [24]. All three genotypes were detected in human samples, with G1 being the most dominant genotype affecting people from Bosnia and Herzegovina (Table 1; Fig. 2). G1H1, G3H2, G7H3, G7H4 and G7H5 were recognized as zoonotic haplotypes (Fig. 2). No insertion mutations or deletions were recorded within the *atp*6 gene fragment for all three genotypes. Overall, the G1 and G3 sequences of *E. granulosus* s.s. individually showed lower haplotype diversities, but greater nucleotide diversities compared to the G7 genotype of *E. canadensis* (Table 2).

The interspecific *p*-distances calculated by the *atp*6 and *cox*1 nucleotide sequences ranged from 0.001 to 0.157 (overall average = 0.086) and 0.000 to 0.089 (overall average = 0.042), respectively, indicating a lower interspecific resolution of the *cox*1 gene. The average intraspecific *p*-distance for the *atp*6 isolates of *E. granulosus* s.s. and *E. canadensis* was 0.005 (range: 0.001–0.009) and 0.003 (range: 0.001–0.004), respectively.

We also observed differences in host preference, tissue tropism and the fertility rate of the identified genotypes, but there was no obvious correlation between the genotypes and their geographical distribution (Fig. 1). For example, G1 showed the highest fertility rate in sheep (*P* = 0.0177) and pigs (*P* = 0.0468), whereas all G3 cysts retrieved from cattle were sterile (*P* = 0.0003). Furthermore, G1 was found to infect the liver of sheep (*P* = 0.0048) and humans (*P* = 0.0222) more frequently than the lungs, similarly to that observed for the G7 genotype in pigs (*P* = 0.0001) (Table 1). G3H1 and G3H2 were unique to wild boars and sheep and cattle, respectively, and all five G7 haplotypes identified in the present study were mostly linked to pig isolates (Fig. 2).

## Discussion

This is the first molecular survey investigating the genetic diversity of *E. granulosus* s.l. in animals and humans in Bosnia and Herzegovina. The *atp*6 data showed that three different genotypes, namely, G1, G3 (*E. granulosus* s.s.) and G7 (*E. canadensis*) circulate among intermediate hosts in the country. Although all three exhibited a low host specificity, G1 was the predominant genotype identified in sheep, G3 in cattle and G7 in pigs. Overall, the most common genotype in our study was G1, with a higher positivity rate of 48.2% compared with the 29.7% reported in Eastern Europe [25]. Our results suggest that the fertility rate and tissue tropism of *E. granulosus* s.l. depend on the genotype and the host species. These findings are consistent with the results of previously reported studies (e.g. [2, 25–29]). Moreover, none of the cattle infected with the *E. granulosus* s.s. G3 genotype displayed fertile CE cysts, which is in agreement with the findings of earlier studies [30–33]. Our result indicates that cattle are not a major intermediate host for the G3 genotype, but rather a dead-end host [34].

Identification of G1, G3, and G7 in human samples indicates the occurrence of a common animal-human transmission pattern, where the farm setting and/or free-roaming dogs seem to play a major role in the transmission ecology. This is principally supported by the fact that home slaughter and the feeding of dogs with the animal offal are still quite common practices throughout Bosnia and Herzegovina. Indeed, active life-cycles of all three genotypes coexist in almost all investigated regions. However, the role played by dogs and wild carnivores in the circulation and transmission of specific genotypes in the country needs to be investigated.

To date, only G1 and G3 from patients originating from Bosnia and Herzegovina have been documented as imported cases in Austria and Slovenia [35, 36], which makes the identification of G7 in this study the first report from the country. Apart from the *Echinococcus* species/genotypes identified in the present study, another zoonotic species, *E. ortleppi* (G5, or cattle strain), has been recently reported in a captive crested porcupine (*Hystrix cristata*) from Sarajevo Zoo [37]. Considering the distribution of this genotype is limited to Central and Western Europe [2], the infection was most likely acquired outside the country, but this is difficult to prove.

The *atp*6 sequence and phylogenetic analyses also revealed an overall low genetic diversity, and this is particularly true for the most common G1 haplotype. The low genetic variability among G1 isolates from Bosnia and Herzegovina may indicate a recent genetic bottleneck event and/or balancing selection [25, 28], which is further supported by the NJ tree structure that displayed only two haplotypes, with the vast majority of sequences belonging to a widely distributed haplotype (G1H1). Bottleneck events have already been recorded for *Echinococcus* isolates [28]. Similarly, low haplotype and nucleotide variations in the *cox*1 gene of *E. granulosus* s.s. (G1 and G3 genotypes) have been reported in animals and humans from other European countries (e.g. [25, 27, 31, 32, 38]), including neighbouring Serbia [28]. The absence of genetic variations between isolates in many European countries could be further explained by the introduction of the ancestral *E. granulosus* s.s. variants through livestock and human migrations from the Middle East [24, 38, 39]. The low genetic variability may also suggest possible genetic exchange between populations of distant geographic areas due to highly mobile intermediate and definitive hosts [27, 38].

In this study, we analysed only one CE cyst per animal/human; consequently, mixed infections with different genotypes/haplotypes cannot be excluded. Mixed infection in a single intermediate host, including humans, has been previously reported, suggesting the outcross breeding of different adult worms in sympatric populations [25, 33, 40–42]. Nevertheless, the concurrent infection could also be the result of a single or successive infection event in the intermediate hosts [43].

## Conclusions

To the best of our knowledge, this is the first systemic study on the genetic diversity of *E. granulosus* s.l. isolates collected from animals and humans in Bosnia and Herzegovina. Our results showed that CE in the country is mainly restricted to *E. granulosus* s.s. (G1 and G3) and *E. canadensis* (G7) clusters. All three identified genotypes (but not all haplotypes) are zoonotic and represent a serious threat to human health. The *atp*6 gene investigated in this study contains useful sites for a proper delineation of G1 and G3 genotypes, and enriched the genetic information on the identified genotypes. In addition, the DNA purification step before PCR may have considerably increased the positivity rate in FFPE tissue samples. Future studies should focus on a wider spectrum of domestic and sylvatic intermediate and definitive hosts to obtain a better insight into the transmission cycles of *E. granulosus* s.l. in Bosnia and Herzegovina. Moreover, we suggest using high-resolution mitochondrial and nuclear markers to advance our understanding of host specificity and the possible zoonotic potential of new haplotypes and haplotypes in general.

## Data Availability

Representative nucleotide sequences were deposited in the GenBank^®^ database and are available under the following accession numbers: OP487713–OP487822 (*atp*6) and OP435590–OP435631 (*cox*1).

## Abbreviations

*atp*6: Adenosine triphosphate 6 gene
CE: Cystic echinococcsis
*cox*1: Cytochrome *c* oxidase 1
FFPE: Formalin-fixed paraffin-embedded
G: Genotype
NJ: Neighbour-joining tree
